# The Common Task

**Published:** 1907-11-09

**Authors:** 


### THE COMMON TASK.

**Correspondence and Queries** for this section should be sent to the Editor of THE HOSPITAL, 28 Southampton Street, Strand, London, and marked "Nursing Administration."

### HOSPITAL BEDDING.

There is little doubt that thousands of pounds are wasted annually in the hospitals over new bedding when, with a good system, the old bedding could be restored and cleaned at small cost. The system introduced by Miss Monk at King's College Hospital years ago has never been improved upon, and might with advantage be copied in institutions which show a heavy outlay year by year on repairs and renewals, an item which in many instances is merely bedding in disguise. Under this plan all bedding, as it is sent away to be cleaned, is weighed, and each mattress, pillow, and bolster is made up to a standard weight found by long experience to be the most satisfactory. The cleaning process consists of washing the tick and thoroughly pulling, purifying, and re-making the hair. Whatever hair is lost in the cleaning or by wear is made good, and the covers are then re-stuffed. Under this system no new mattresses, bolsters, or pillows are ever needed unless the number of beds is added to, the sole outlay being for the labour and for extra hair needed in the process. A competent upholstress is a necessary and most economical adjunct to a large hospital, and even when there is not sufficient work to keep one constantly employed, it will be found more economical to engage one by the day to execute work on the premises rather than send such cleaning and repairs as the above to be done at a shop. The reason for this is that the shop has every interest in not making use of old materials, and that the hospital gets the full benefit of such savings when the work can be supervised by those who have a strong motive for economy.

### THE MATRON'S AUTHORITY.

Why is it considered so essential by those who know that nurses shall be placed under the control of the matron in preference to that of the medical superintendent? For the same reason that it is the mistress of the house and not the master who invariably controls the female staff. Women in training for nurses need the supervision and discipline which can only be rightly exercised by one of their own sex. And if the authority is to be effectual there must be no court of appeal to another person in the same household. The law of the land which provides that agreements once entered upon shall be faithfully carried out by both parties is sufficient guarantee that no serious injustice will be inflicted on those thus subordinated to the matron. To introduce an arbitrator in the person of the medical superintendent is to place both matron and doctor in an exceedingly awkward position. Imagine a private household in which the maids knew that they might appeal from the decisions of their mistress! It could hardly have any other result than the breaking up of the home. If this over-lordship of the matron were lodged in the hands of some lady of the governing body it would still be bad, but would be less likely to be destructive of good feeling than lodging the ultimate authority in the hands of a person of the opposite sex. For it is a curious fact that although women often attain to great skill in training and ruling men in subordinate positions, the same hardly ever holds good with regard to men set in authority over women. The best administrators frankly admit this, and are among the most determined advocates for the supremacy of the matron in her own domain.

